# Multimodel inference for biomarker development: an application to schizophrenia

**DOI:** 10.1038/s41398-019-0419-4

**Published:** 2019-02-11

**Authors:** Jason D. Cooper, Sung Yeon Sarah Han, Jakub Tomasik, Sureyya Ozcan, Nitin Rustogi, Nico J. M. van Beveren, F. Markus Leweke, Sabine Bahn

**Affiliations:** 1 Department of Chemical Engineering and Biotechnology, University of Cambridge, Cambridge, UK; 2 Department of Neuroscience, Erasmus Medical Centre, Rotterdam, Netherlands; 3 Department of Psychiatry, Erasmus Medical Centre, Rotterdam, Netherlands; 4 Department “Nieuwe Kennis”, Delta Centre, for Mental Health Care, Rotterdam, Netherlands; 5 Brain and Mind Centre, University of Sydney, Sydney, Australia; 6 Present Address: Department of Chemistry, Middle East Technical University, Ankara, Turkey

## Abstract

In the present study, to improve the predictive performance of a model and its reproducibility when applied to an independent data set, we investigated the use of multimodel inference to predict the probability of having a complex psychiatric disorder. We formed training and test sets using proteomic data (147 peptides from 77 proteins) from two-independent collections of first-onset drug-naive schizophrenia patients and controls. A set of prediction models was produced by applying lasso regression with repeated tenfold cross-validation to the training set. We used feature extraction and model averaging across the set of models to form two prediction models. The resulting models clearly demonstrated the utility of a multimodel based approach to make good (training set AUC > 0.80) and reproducible predictions (test set AUC > 0.80) for the probability of having schizophrenia. Moreover, we identified four proteins (five peptides) whose effect on the probability of having schizophrenia was modified by sex, one of which was a novel potential biomarker of schizophrenia, foetal haemoglobin. The evidence of effect modification suggests that future schizophrenia studies should be conducted in males and females separately. Future biomarker studies should consider adopting a multimodel approach and going beyond the main effects of features.

## Introduction

Despite our ever increasing ability to generate data, many published findings are not reproducible in independent data sets^1–3^. In biological psychiatry, this situation is further exaggerated by the lack of a biological ‘gold standard’ diagnoses for psychiatric disorders^4,5^, which are still diagnosed based on the evaluation of signs and symptoms in clinical interviews. One notable limitation of symptom-based diagnosis is that the boundaries between disorders can be poorly defined because of overlapping symptoms and common co-morbidity across psychiatric disorders, which can result in a layering or commingling of symptoms^6^. Consequently, patient groups are biologically heterogenous^4^, misdiagnosis is common^7^ and prediction models attempt to link biological data to a symptom-based diagnosis^4,5^.

Nevertheless, although many significant psychiatric disorder biomarker findings have been reported, only few have been consistently replicated^8,9^. This lack of reproducibility is a result of underpowered replication studies for the small to moderate effect sizes initially reported^3,5^, differences in patient selection criteria^5^ (e.g. age, recency of diagnosis, sex ratio^10^, treatment, and comorbidities) and inconsistencies in methods used to quantify biological markers. In addition, when model selection is performed in high-dimensional data, defined as data with more features (variables) than subjects, overfitting a model can be a major issue and be compounded by biologically heterogeneous psychiatric patient populations. Overfitting occurs when the coefficient estimates of the selected model depend not only on the underlying relationship of interest, but also on chance characteristics of the data analysed. When an overfitted model is applied to new subjects the predictive performance is reduced. In other words, the model provides an over-optimistic assessment of the predictive performance when based on the data to which the model was fitted.

Despite the heterogeneity of psychiatric patients, models with a more reproducible predictive performance can be achieved by taking into account model selection uncertainty. Model selection is often considered to be a process of selecting a single model from a set of all possible models that is judged to be the ‘best’ model for making inferences from the analysed data^11,12^. Any uncertainty in model selection, for example, resulting from a small change in the data set, is ignored once the best model has been found. In the present study, rather than making predictions based on a single-best model selected from a data set, we adopted a multimodel approach to make predictions for the probability of having schizophrenia based on a set of models to allow for any uncertainty in model selection. The schizophrenia data analysed consisted of two-independent mass spectrometry (MS) multiple reaction monitoring (MRM) proteomic data sets (147 peptides from 77 proteins) of first-onset drug-naive schizophrenia patients and controls^13,14^ that were used in the present study as training and test sets. The sensitivity of model selection to small changes in the training data was evaluated by model selection using least absolute shrinkage and selection operator (lasso) regression with the resampling approach of repeated tenfold cross-validation^15^. In the absence of one model being superior to the other selected models, we used feature extraction and model averaging across the set of models to form the prediction model. This approach improved the generalizability of the model, that is, it reduced overfitting and provided more reproducible inference. We then attempted to validate the model predictive performance by applying the model to an independent test set.

## Materials and methods

### Subjects

The Cologne study (referred to as the ‘training set’), as previously described^13^, consisted of serum samples from 60 first-onset drug-naive schizophrenia patients and 79 age and sex matched controls recruited by the Department of Psychiatry, University of Cologne (Supplementary Table 1). The Rotterdam study (referred to as the ‘independent test set’), as previously described^14^, consisted of nine first-onset drug-naive male schizophrenia patients and 12 male controls recruited by the Erasmus Medical Centre in Rotterdam (Supplementary Table 1). Schizophrenia was diagnosed based on the Diagnostic and Statistical Manual of Mental Disorders IV (DSM-IV)^16^. The ethical committees of the medical faculties of the respective universities approved the protocols of the study. Informed consent was given in writing by all participants and clinical investigations were conducted according to the Declaration of Helsinki.

### Targeted protein quantification

Serum samples were prepared in a 96-well plate format as described previously^17^. Briefly, serum samples were diluted with ammonium bicarbonate. Then, disulphide bond reduction and cysteine alkylation were performed using Dithiothreitol (DTT) and Iodoacetamide (IAA), respectively. Proteins were digested overnight using trypsin (see Supplementary Information). Isotopically labelled internal standard peptides were spiked into serum samples prior to MS run. Quality control (QC) samples were used in this study to monitor method performance and instrument stability (see Supplementary Information).

In this study, a total of 101 proteins (172 peptides), the majority previously associated with psychiatric disorders, were selected. Three to four interference free transitions were selected for each targeted peptide as described previously^17^. Tryptic digested peptides were monitored using an Agilent 1290 liquid chromatography (LC) system coupled with 6495 Triple Quadrupole mass spectrometer equipped with jet-stream nano ESI source operated in positive mode. MS data were acquired in MRM mode. The chromatographic separation was carried out on Agilent AdvanceBio Peptide Map column (2.1 × 150 mm 2.7-micron) at 50 °C. Peptides were eluted over a linear gradient from 3 to 30% acetonitrile in 0.1% formic acid in 45 min.

### Statistical analysis

#### Data pre-processing and quality control

We processed raw mass spectrometry (MS) files using the Skyline software package (Version 3.1.0)^18^. We manually checked peaks and when necessary, adjusted peak integrations accordingly. The endogenous and internal standard peptide-transition peak areas were estimated and exported as a comma delimited data file for statistical analysis in R (Version 3.4.4)^19^. The MS data pre-processing is described in Supplementary Information.

#### Model selection

We used lasso regression with repeated tenfold cross-validation to reduce overfitting and to investigate model selection uncertainty in the training set.

#### Tenfold cross-validation

Tenfold cross-validation is a commonly used resampling approach to reduce the problem of overfitting^11^. The data are randomly split into tenfolds. We hold out each fold one at a time, train on the remaining data and predict the held out observations for each value of the regularization parameter – selecting the regularization parameter that minimises the cross-validation deviance (classification error). The model, as defined by the regularization parameter, is then fit to the entire data set^11^. We repeatedly applied tenfold cross-validation 100 times to determine how sensitive model selection was to small changes in the training set (overfitting). Note that changes in the training set result from the data being randomly split into tenfolds for each application of tenfold cross-validation.

#### Lasso regression

Lasso regression is a penalized regression approach that reduces overfitting by placing a constraint on the sum of the absolute values of the regression coefficients, which shrinks the coefficients, a process referred to as regularization or shrinkage, and allows poor predictors to be shrunken exactly to zero (variable selection)^20^. Shrinkage often improves the prediction accuracy^11^. The constraint (also known as the regularization parameter, shrinkage parameter or penalty) was selected using tenfold cross-validation. Lasso regression with tenfold cross-validation was conducted using the R package glmnet^15,20^. We set the elastic-net penalty, *α*, that bridges the gap between lasso (*α* = 1, the default) and ridge regression (*α* = 0), to 0.9 for numerical stability^15,20^.

#### Akaike information criterion

We adopted a model averaging approach using the Akaike information criterion (AIC) weights as described in Burnham and Anderson^11,12^. We calculated the AIC for each model selected by lasso regression with tenfold cross-validation. The AIC is a measure of how well a model fits the data relative to the other possible models given the data analysed and favours fewer parameters^21^. The model with the lowest AIC is the best model approximating the outcome of interest. AIC can be expressed as:

$${\rm AIC} = - 2({\rm log}\,{\rm likelihood}) + 2K,$$

where *K* = number of model parameters and log-likelihood is a measure of model fit^12^. In this study, as *n*/*K* ≤ 40 for sample size *n* and the model with the largest value of *K*, we used the second-order bias correction version of the AIC:

$${\rm AIC}_c = - 2({\rm log}\,{\rm likelihood}) + 2K + \frac{{2K(K + 1)}}{{n - K - 1}},$$

$${\rm AIC}_c = {\rm AIC} + \frac{{2K(K + 1)}}{{n - K - 1}},$$

where *n* = sample size, *K* = number of model parameters and log-likelihood is a measure of model fit^12^.

#### Akaike weights

After model selection, we calculated the Akaike weights, *w*_*m*_, for each model:

$$w_m = \frac{{{\mathrm{exp}}\left( { - \frac{1}{2}{\rm AIC}_m} \right)}}{{\mathop {\sum }\nolimits_{j = 1}^M {\mathrm{exp}}\left( { - \frac{1}{2}{\rm AIC}_j} \right)}},$$

where *w*_*m*_ and AIC_*m*_ are, respectively, the Akaike weight and AIC_*c*_ for model *m* and AIC_*j*_ is the AIC_*c*_ for model *j* *=* 1 to *M*. The denominator normalizes the Akaike weights, so that

$$\mathop {\sum }\limits_{m = 1}^M w_m = 1.$$

The Akaike weights can be interpreted as model probabilities or the ‘weight of evidence’ in favour of model *m* being the best model based on the available data set^12^. The Akaike weights can be used to quantify the evidence for the importance of each feature in the set of selected models^11^. The relative importance of feature *f* is the summation of the Akaike weights across the set of selected models which include feature *f*^11^. The resulting relative feature importance, between 0 and 1, allows features to be ranked by their importance and can be interpreted as the probability of the feature being included in the best model for the data. The relative importance of subsets of features occurring together can also be quantified^11^.

#### Feature extraction

We defined two prediction models, the first based the frequency of feature selection (inclusion fraction > 0.80) and the second based on the probability that a feature is included in the best model for the data (inclusion probability > 0.90). Both models have the added advantage of further reducing overfitting by excluding features less frequently selected or with low inclusion probabilities (Akaike weights). We note that both the inclusion fraction and probability thresholds were determined before the analysis was conducted.

#### Model averaging

If one of the selected models was clearly superior to the other selected models [for example, it has a probability that it is the best model for the data, *w* > 0.9^11^], then inference could be based on that model alone. However, when model selection uncertainty is evident, inference based on a set of models can result in more reproducible inference. To obtain more reproducible predictions of schizophrenia diagnosis, we adopted a model averaging approach, specifically, for a feature of interest, we estimated their weighted average coefficient, $\widehat {\bar \beta }$, across a set of models derived from applying lasso regression with repeated tenfold cross-validation to the training data set.

$\widehat {\bar \beta } = \mathop {\sum }\limits_{j = 1}^M w_j\hat \beta _j$, where *w*_*j*_ and $\hat \beta _j$ are, respectively, the Akaike weight and coefficient estimates for a feature of interest in model *j* and $\widehat {\bar \beta }$ is the weighted average of $\hat \beta _j$ over models *j* *=* 1 to *M*^11^. As all models are considered, those models not containing the feature of interest contribute zero to the weighted average estimate which results in the coefficient shrinking towards zero.

#### Model selection including first-order interactions with sex

As the two prediction models had a substantially better predictive performance for males than for females (Table 2a), we wanted to investigate whether the effect of protein abundance on the probability of having schizophrenia was modified by the sex of the individual. To investigate effect modification, we needed to consider first-order interactions between protein abundance and sex in model selection. If we consider a simple logistic model

$${\rm logit}\left( \pi \right) = \alpha + \beta _1X_1 + \beta _2X_2,$$

where Pr(*Y* = 1) = *π*, logit(*π*) is the logit function of this probability [natural log of *π*/(1 – *π*)], *α* is the intercept and, *β*_1_ and *β*_2_ are coefficients for features *X*_1_ and *X*_2_. Typically, a product term *X*_1_*X*_2_ is added to the model to allow for an interaction

$${\rm logit}\left( \pi \right) = \alpha + \beta _1X_1 + \beta _2X_2 + \beta _3X_1X_2.$$

The coefficient of the product term, *β*_3_, reflects interaction as a departure from the multiplicative effects, in other words, the combined effect of *X*_1_ and *X*_2_ is larger (or smaller) than the product of their individual main effects.

To consider interactions between protein abundance and sex in the model selection with repeated tenfold cross-validation, we adopted the glinternet approach of Lim and Hastie^22^. Glinternet is based on group lasso^23^ and importantly, ensures hierarchically well formulated models, that is, an interaction can only be selected if both of its main effects are selected^22^. In other words, if an interaction term is selected and main effects have not, the main effects will also be selected. We used the R package glinternet^22^.

#### Predictive performance

Predictive performance was evaluated using the area under the receiver operating characteristic (ROC) curve. The area under the curve (AUC) measures the extent to which a model’s predicted probability agrees with the observed outcome, that is the presence or absence of an event. The AUC is the probability that a randomly chosen patient with the event is rated/ranked higher than a randomly chosen patient without the event. A model performing no better than random will have an AUC of 0.50. (AUC: 0.9–1 = excellent; 0.8–0.9 = good; 0.7–0.8 = fair; 0.6–0.7 = poor; 0.5–0.6 = fail). The AUC was calculated using the R package ROCR^24^.

#### Pathway analysis

Biological process pathway analysis was carried out using Gene Ontology and PANTHER^25^. UniProt accession numbers of proteins corresponding to the peptides selected in the final model were uploaded to http://geneontology.org and all Homo sapiens genes in the database were used as a reference list. Fisher’s exact with false discovery rate (FDR) multiple test correction was used for determining pathway significance.

## Results

After MS data pre-processing, there were 77 proteins (147 peptides) measured in a training set of 60 first-onset drug-naive schizophrenia patients and 77 controls, and in a male-only independent test set of nine first-onset drug-naive schizophrenia patients and 12 controls (Supplementary Table 1).

### Model selection

**Fig. 1:**
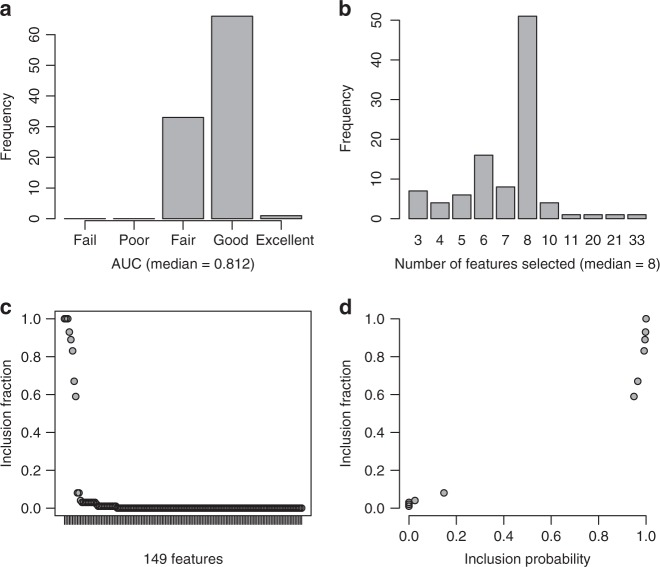
A summary of the 100 models selected using lasso regression with repeated cross-validation. **a** A bar chart summarizing the AUCs for each model. AUC: Fail 0.5–0.6; Poor 0.6–0.7; Fair 0.7–0.8; Good 0.8–0.9; and, 0.9–1.00 Excellent. **b** A bar chart summarizing the number of features selected in each model. **c** An inclusion fraction plot summarizing the proportion of times each feature was selected in a model. One hundred and seventeen features out of 149 (147 peptides, sex, and age) were not selected in the 100 models. **d** A plot of inclusion frequencies and probabilities for the 32 selected features

Model selection using lasso regression with repeated tenfold cross-validation revealed some uncertainty in model selection in the training set (Fig. 1). Based on the features selected, there were 11 unique models (Supplementary Table 2) with, on average, a good predictive performance (AUC = 0.81; Fig. 1a). The number of features selected ranged from 3 to 33, with eight on average (Fig. 1b). Although 33 features out of 149 (147 peptides, sex, and age) were selected at least once (Fig. 1c; Supplementary Table 3), 25 of these features were selected less than 10 times out of 100, consistent with limited model overfitting. The remaining eight features were selected more than 50 times, six of which were selected more than 80 times out of 100 (Table 1). There was not a single best model approximating schizophrenia status in the training set; the most frequently selected model had eight features and was selected 51 times out of 100.

**Table 1:** A summary of the model averaged coefficients for the two models, the first consisting of six features with an inclusion fraction >0.8 and the second consisting of eight features with an inclusion probability (relative feature importance) >0.9

		Inclusion fraction	Inclusion probability	Mean coefficient	Weighted mean coefficient	Model
(Intercept)		–	–	0.792	1.027	1, 2
APOA4	IDQNVEELK	1.00	1.000	−0.238	−0.320	1, 2
APOC3	GWVTDGFSSLK	1.00	1.000	−0.287	−0.334	1, 2
HPT	VTSIQDWVQK	1.00	0.998	0.266	0.287	1, 2
IC1	TNLESILSYPK	0.93	0.996	0.141	0.196	1, 2
APOA2	SPELQAEAK	0.89	1.000	−0.248	−0.390	1, 2
ITIH4	GPDVLTATVSGK	0.83	0.992	0.0932	0.153	1, 2
ANT3	LPGIVAEGR	0.67	0.965	0.0382	0.0775	2
APOH	EHSSLAFWK	0.59	0.949	0.0320	0.0622	2

The mean coefficient is the mean of the coefficients for the feature of interest based on all of the models. The weights used for the weighted mean coefficient are the model probabilities (Akaike weights)

### Akaike model weights

**Fig. 2:**
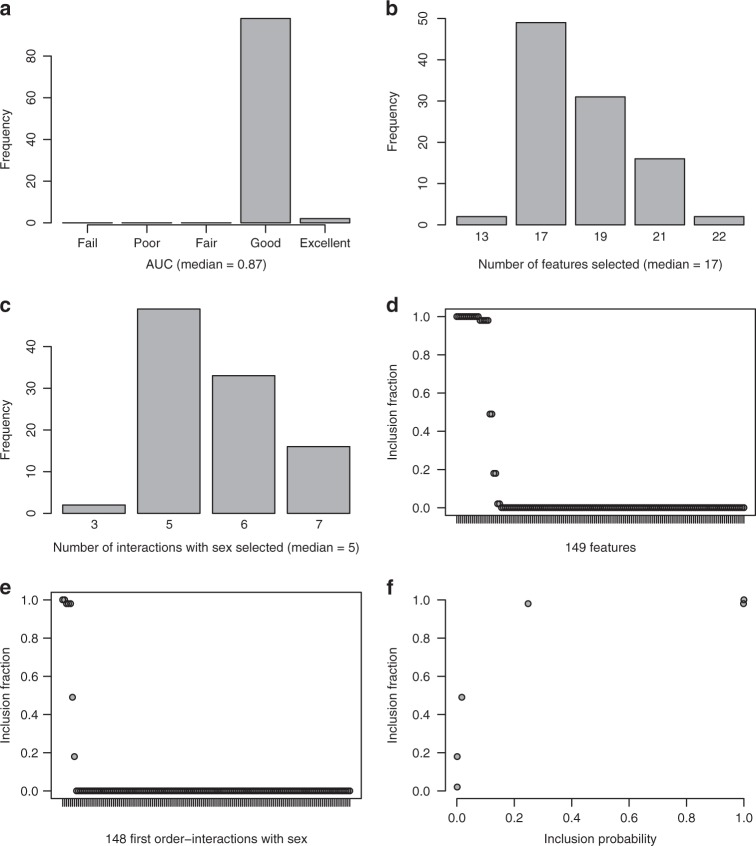
A summary of the 100 models selected using glinternet with repeated cross-validation. **a** A bar chart summarizing the AUCs for each model. AUC: Fail 0.5–0.6; Poor 0.6–0.7; Fair 0.7–0.8; Good 0.8–0.9; and, 0.9–1.00 Excellent. **b** A bar chart summarizing the number of features selected in each model. **c** A bar chart summarizing the number of first-order interactions with sex selected in each model. **d** An inclusion fraction plot summarizing the proportion of times each feature was selected in a model. **e** An inclusion fraction plot summarizing the proportion of times each first-order interactions with sex was selected in a model. One hundred and twenty six features out of 149 (147 peptides, sex, and age) were not selected in the 100 models. **f** A plot of inclusion frequencies and probabilities for the selected features and interactions

Akaike weights were calculated for each of the 100 selected models. As there were 11 unique models, we summed the weights by each unique model to estimate the probability of it being the best model approximating the probability of having schizophrenia in the training set. Despite some uncertainty in model selection, the three models with the highest probability of being the best model had a combined probability of over 0.95; five models had a combined probability of over 0.99 (Supplementary Table 2). The most frequently selected model had the highest single model probability of 0.80. We note that frequency of selection does not necessarily equate to the probability of being the best model for the data analysed (Figs 1d and 2f; Supplementary Tables 1 and 3).

### Model averaging

We defined two models using feature extraction (Table 1), the first consisting of six features with an inclusion fraction >0.8 and the second consisting of eight features with an inclusion probability (relative feature importance) >0.9. Model averaging for each feature of interest was conducted across all 100 selected models. After model averaging, the first model (six features) was applied to the training set and then to the independent test set, both had a good predictive performance, AUC of 0.81 and 0.88, respectively (Table 2a). A similar predictive performance was obtained when the second model (eight features) was applied to the training set and then to the independent test set, AUC of 0.82 and 0.92, respectively (Table 2a).

**Table 2:** A summary of predictive performance in the training and independent test sets of the prediction models with averaged coefficients

	Training set (Cologne)		Independent test set (Rotterdam)
Schizophrenia patients	60		9
Controls	77		12
(a)	Training set		Independent test set
	Number of features	AUC	AUC
Model with >0.80 inclusion fraction	6	0.807 Males only 0.851 Females only 0.751	Males only 0.880
Model with relative feature importance >0.90	8	0.821 Males only 0.863 Females only 0.773	Males only 0.917
(b)			
Model with >0.80 inclusion fraction	17 with 5 first-order interactions with sex	0.858 Males only 0.890 Females only 0.819	Males only 0.889
Model with relative feature importance >0.90	13 with 3 first-order interactions with sex	0.854 Males only 0.887 Females only 0.818	Males only 0.815

Model selection (a) did not consider first-order interactions with sex and (b) did allow for first-order interactions with sex

To investigate the higher predictive performance in the male-only independent test set, we then applied the two models to training set males and females separately. We found that the predictive performance was substantially higher for males than for females (Table 2a). We note that sex had not been selected in any of the 100 models (Supplementary Tables 1 and 2) and that first-order interactions between features and sex had not been considered in the model selection.

### Model selection considering first-order interactions with sex

To investigate potential differences between males and females, we adopted the glinternet approach of Lim and Hastie^22^ to consider first-order interactions between protein abundance and sex in model selection with repeated tenfold cross-validation. Importantly, this approach ensures that the model obeys strong hierarchy, that is, if an interaction is selected, both of its main effects will also be selected^22,26^. We only considered first-order interactions with sex.

After considering interactions in model selection, there was less uncertainty in model selection. Based on the features selected, there were five unique models (Supplementary Table 4) with, on average, a good predictive performance (AUC = 0.87; Fig. 2a). Notably, all five of the unique models included interaction terms. The vast majority of selected features (19/23; Fig. 2d) and interactions (5/7; Fig. 2e) were selected at least 98 times out of 100. The two APOC3 peptides were selected in every model despite a strong linear relationship between them (correlation coefficient *r* = 0.87 and *P* < 2.2 × 10^−16^). The eight features selected in the earlier analysis (Table 1) were selected in every model, but none had interactions with sex selected (Supplementary Table 4). The five frequently selected interactions suggest that the sex of a subject was modifying the effects of peptides from APOE, A2AP, HBA, HBG1, and SHBG on the probability of having schizophrenia. The difference between males and females in the abundance of these five peptides in schizophrenia patients and controls is shown in Supplementary Figure 3. Consequently, model selection should be conducted in males and females separately to allow for biological differences between the sexes. However, in the present study, the training set was too small to conduct model selection in males and females separately.

As in the previous analysis, we defined two models (Table 3), the first consisting of 17 features and five interactions with an inclusion fraction of >0.8 and the second consisting of 13 features and three interactions with an inclusion probability >0.9. Model averaging for each feature of interest was conducted across all 100 selected models. After model averaging, the first model was applied to the training set and then to the independent test set. The marked difference seen in the previous analysis was no longer evident and both had a good predictive performance, AUC of 0.86 and 0.89, respectively, demonstrating the reproducibility of the model (Table 2b). A similar predictive performance was obtained when the second model was applied to the training set and then to the independent test set, AUC of 0.85 and 0.82, respectively (Table 2b). Despite the good predictive performance and the reproducibility, when we applied the two models to training set males and females separately, although marginally less than in the previous analysis, the predictive performance remained substantially better for males than for females (Table 2b).

**Table 3:** A summary of the model averaged coefficients for the two models, the first consisting of 17 features and five interactions with an inclusion fraction of >0.8 and the second consisting of 13 features and three interactions with an inclusion probability (relative feature importance) >0.9

UniProt accession number^27^	Main effects		Inclusion fraction	Inclusion probability	Mean coefficient	Weighted mean coefficient	Model
	(Intercept)		–	–	1.5400	1.1300	1, 2
	Female		1.00	1.000	0.0356	0.01180	1, 2
**P00738**	**HPT**	**VTSIQDWVQK**	1.00	1.000	0.2800	0.28100	1, 2
**P05155**	**IC1**	**TNLESILSYPK**	1.00	1.000	0.1720	0.15700	1, 2
**Q14624**	**ITIH4**	**GPDVLTATVSGK**	1.00	1.000	0.2390	0.17600	1, 2
P04278	SHBG	IALGGLLFPASNLR	1.00	1.000	0.1260	0.08490	1, 2
**P01008**	**ANT3**	**LPGIVAEGR**	1.00	1.000	0.0540	0.04700	1, 2
**P02652**	**APOA2**	**SPELQAEAK**	1.00	1.000	−0.4380	−0.35800	1, 2
**P06727**	**APOA4**	**IDQNVEELK**	1.00	1.000	−0.4580	−0.38300	1, 2
**P02656**	**APOC3**	**GWVTDGFSSLK**	1.00	1.000	−0.3240	−0.33900	1, 2
P02656	APOC3	DALSSVQESQVAQQAR	1.00	1.000	−0.0998	−0.04730	1, 2
P02649	APOE	LEEQAQQIR	1.00	1.000	−0.1920	−0.12900	1, 2
**P02749**	**APOH**	**EHSSLAFWK**	1.00	1.000	0.1990	0.12900	1, 2
P08697	A2AP	DFLQSLK	0.98	0.999	−0.0269	−0.03220	1, 2
O75636	FCN3	YGIDWASGR	0.98	0.248	0.0552	0.01090	1
P02765	FETUA	HTLNQIDEVK	0.98	0.248	−0.1120	−0.02180	1
P69905	HBA	MFLSFPTTK	0.98	0.248	−0.0120	−0.00228	1
P69891	HBG1	MVTAVASALSSR	0.98	0.248	−0.0101	−0.00200	1
*First-order interactions with sex*							
P04278	SHBG	IALGGLLFPASNLR	1.00	1.000	−0.3180	−0.21100	1, 2
P02649	APOE	LEEQAQQIR	1.00	1.000	0.5510	0.37700	1, 2
P08697	A2AP	DFLQSLK	0.98	0.999	0.0805	0.09690	1, 2
P69905	HBA	MFLSFPTTK	0.98	0.248	0.0514	0.00930	1
P69891	HBG1	MVTAVASALSSR	0.98	0.248	0.0425	0.00817	1

The mean coefficient is the mean of the coefficients for the feature of interest based on all of the models. The weights used for the weighted mean coefficient are the model probabilities (Akaike weights). The eight features selected in the earlier analysis (Table 1), are shown in bold. HBA and HBG1 are haemoglobin subunits alpha and gamma-1, respectively

## Discussion

Seventy-seven proteins (147 peptides) were measured in serum samples from two-independent collections of first-onset drug-naive schizophrenia patients and matched controls using mass spectrometry. We were able to demonstrate that multimodel inference can provide models for the probability of having schizophrenia with a good (training set AUC > 0.8) and reproducible predictive performance (test set AUC > 0.8; Table 2). A notable advantage of the multimodel approach, particularly evident from the set of selected models (Supplementary Tables 1 and 3), is that it reduces the risk of selecting one of the less probable models by chance. In addition, feature extraction using the inclusion fractions or probabilities to select features for the model has the effect of further limiting model overfitting. Although we used both inclusion fractions and probabilities for feature selection, the latter is a more sensible approach as it represents how likely each feature is to be included in the best model for the data analysed.

After observing the differences in the prediction performance between males and females in the training set (Table 2a), extending the analysis to include first-order interactions was essential to determine whether the effect of protein abundance on the probability of having schizophrenia was modified by sex. As the vast majority of the selected models contained the same five interactions (Supplementary Table 4), there was sufficient evidence of modification by sex, suggesting that future schizophrenia biomarker studies should ideally be conducted in males and females separately. This would allow for biological differences underpinning the reported sex differences in schizophrenia to be better utilized in the prediction model. Reported sex differences include males having an earlier onset, more negative and less depressive symptoms while females experience more emotional and psychotic symptoms^28,29^.

Despite concerns about the impact of symptom-based diagnosis of schizophrenia on model selection uncertainty, resampling using repeated tenfold cross-validation revealed a large degree of stability in the features selected (Table 2; Fig. 1c) that was notably improved with the inclusion of first-order interactions with sex (Table 3; Fig. 2d, e). The similar participant selection criteria, particularly the recruitment of first-onset drug-naive schizophrenia patients, in the training and independent test sets would have contributed to this stability (Supplementary Table 1; see Methods).

As 30 of the 77 proteins analysed have been previously associated with schizophrenia (Supplementary Table 6), it was re-assuring, although not unexpected, that the majority of the selected proteins (12 of 14; Table 3) have been previously associated with schizophrenia^30–33^ with the most robust finding for increased HPT levels in schizophrenia patients compared to controls^30,34–37^. In addition, the most significant pathways related to the selected proteins, namely altered acute inflammatory response [involving ANT3, APOA2, A2AP, FETUA, HPT, and ITIH4; gene ontology (GO) term GO:0002526, false discovery rate (FDR) = 1.6 × 10^–7^] and ultra-low density lipoprotein transport (APOA2, APOA4, APOC3 and APOE; GO:0034378, GO:0034371 and GO:0034370, FDR < 1.0 × 10^−6^) in schizophrenia, are consistent with previous reports^36,38^. The fact that proteins from these pathways also correlate with disease psychopathology scores, in particular-negative symptoms^39–41^, suggests that these processes might mechanistically underpin certain aspects of schizophrenia pathophysiology and as such constitute promising drug targets for add-on treatments.

Model selection using glinternet to allow for first-order interactions identified five peptides from APOE, A2AP, HBA, HBG1 and SHBG (Table 3) whose effect on the probability of having schizophrenia was modified by sex. Interestingly, neither sex nor any of the five protein peptides were selected in the initial model selections that did not consider interactions (Supplementary Table 3). While APOE, A2AP, and SHBG have been previously implicated in schizophrenia^33,42,43^, the association with HBA and HBG1 has not been reported before. HBA and HBG1 are haemoglobin subunits alpha and gamma-1, respectively. Although subunit alpha may belong to different types of haemoglobin (e.g. A, A_2_ and F), the selection of peptides from subunits alpha and gamma-1 by the algorithm, with the latter subunit specific to foetal haemoglobin (haemoglobin F or haemoglobin *α*_2_*γ*_2_), as well as a moderate linear relationship between the subunits (*r* *=* 0.57 and *P* = 2.0 × 10^−7^), indicate that this findings may represent foetal haemoglobin^44^.

There are several limitations to the present study. First, the independent training and test sets had a relatively small sample size with limited demographic and clinical data measured. Second, although a male-only independent test set was not ideal, it proved to be insightful with regard to the sex differences in the protein effects on the probability of having schizophrenia. Had the independent test set consisted of males and females, the reproducibility of the prediction model would have depended on how close the sex ratio was between the two data sets and the sex differences would have been less evident. Finally, we have fitted models to distinguish patients from controls, although this may not reflect the relevant clinical population for a schizophrenia diagnostic test, it is an appropriate first step towards distinguishing between individuals at an ultra-high risk of developing schizophrenia who do and do not develop the disorder over a set time period.

In conclusion, we have demonstrated the utility of a multimodel-based approach to make good and reproducible predictions for a complex psychiatric disorder. We have also demonstrated the importance of considering first-order interactions in model selection and propose that future biomarker studies of schizophrenia should be conducted in males and females separately.

## Supplementary information

Supplementary Material.

## Data Availability

The data sets analysed during the current study are available from the corresponding author on reasonable request.
