# Supplementary material for: Multimodel inference for biomarker development: an application to schizophrenia
Source: Transl Psychiatry. 2019 Feb 11;9:83. doi: 10.1038/s41398-019-0419-4 (PMC6370882; doi:10.1038/s41398-019-0419-4)
Supplement: Supplementary file 1 — Supplementary Material. [file 41398_2019_419_MOESM1_ESM.docx]

Supplementary Information

1. Supplementary methods

1.1 Serum protein sample preparation

For Cologne and Rotterdam samples, 5ul aliquots of serum samples were added to 50mM ammonium bicarbonate followed by disulphide bond reduction and cysteine alkylation using 32.5mM Dithiothreitol (DTT) and 75mM Iodoacetamide (IAA), respectively, and digested overnight with 1:50 (w/w) trypsin. Isotopically labelled internal standard peptides were spiked into the serum samples prior to the mass spectrometry (MS) run.

The Cologne samples were randomised to allocate equal numbers of patients and controls, and males and females to two plates. The Rotterdam samples along with samples from another small study were randomised to one plate. The researchers conducting the sample preparation and MS analysis were blinded to the disease status of the samples.

1.2 Quality control samples

Two quality control (QC) samples were used to monitor method performance and instrument stability. We used commercial serum (Human Sera S7023, Sigma Aldrich) as a QC sample, which was aliquoted, digested and run separately to monitor sample preparation variation. Cologne and Rotterdam pooled QC samples were prepared for by pooling equal volumes of digested samples randomly assigned to plate 1. A corresponding pooled sample was then aliquoted and run once every day alongside the clinical samples during the total experimental run.

1.3 Statistical quality control

In the Cologne MS data, we selected the most abundant peptide-transitions with 80% consistency across MS runs between the endogenous and internal standard, and for the most abundant peptide-transitions with less consistency, we visually checked the peptide for interference from the matrix and manually selected the most abundant transition based on the pooled plate samples. In addition, we applied a relative abundance ratio filter of 1:10 to 10:1, importantly, calculated on the original scale of measurement (for more details see ^1^). We then applied the Cologne peptide-transition selection to the Rotterdam MS data. After abundance ratio filter exclusions, 77 proteins (147 peptides) were available for analysis in the Cologne and Rotterdam studies (see Supplementary Table 5).

We used principal component analysis (PCA) to identify any outliers based on their relative peptide abundance. Two controls from Cologne were identified as outliers and excluded; the remaining 77 controls were analysed (see Supplementary Table 1 and Supplementary Figure 1). The median coefficient of variation was 5.01% [5.49% in plate 1 (seven pooled samples) and 4.53% in plate2 (four pooled samples); Supplementary Figure 2] for Cologne and 5.36% (6 pooled samples in plate 1) for Rotterdam based upon 77 proteins (147 peptides).

Normalisation

We performed normalisation based on the internal standard to minimize non-biological, systematic variation (technical variation) across MS runs. We used the peak area ratio to normalise the data, that is, the abundance ratio of the endogenous peptide-transitions to their internal standard.

Variance stabilisation

As the variance of biological measurements often increases with intensity, we applied a log_2_ transformation, which is commonly used as a variance stabilising transformation as the variation of the logged abundances is less dependent on the absolute magnitude; skewed distributions become more symmetric and the influence of high-abundance transitions when analysed is reduced ^2^.

Coefficient of variation

We used the geometric coefficient of variation (CV), which describes the amount of variability relative to the mean, to quantify the degree of variation for the peptides across the MS runs. For natural log transformed data, the geometric CV = $\sqrt{e^{{sd}^{2}}-1}\times100$ (ref ^3^), where sd is the standard deviation of the log-transformed data. Note that the geometric CV was used as it is important to estimate the variability on the original scale of measurement.

1.4 Model selection

*Ten-fold cross-validation*

Ten-fold cross-validation is a commonly used resampling approach to reduce the problem of overfitting ^11^. The data are randomly split into ten-folds. We hold out each fold one at a time, train on the remaining data and predict the held out observations for each value of the regularization parameter – selecting the regularization parameter that minimises the cross-validation deviance (classification error). The model, as defined by the regularization parameter, is then fit to the entire dataset ^11^. We repeatedly applied ten-fold cross-validation 100 times to determine how sensitive model selection was to small changes in the training set (overfitting). Note that changes in the training set result from the data being randomly split into ten-folds for each application of ten-fold cross-validation.

*Lasso regression*

Lasso regression is a penalized regression approach that reduces overfitting by placing a constraint on the sum of the absolute values of the regression coefficients, which shrinks the coefficients, a process referred to as regularization or shrinkage, and allows poor predictors to be shrunken exactly to zero (variable selection) ^4^. Shrinkage often improves the prediction accuracy ^11^. The constraint (also known as the regularization parameter, shrinkage parameter or penalty) was selected using ten-fold cross-validation. Lasso regression with ten-fold cross-validation was conducted using the R package glmnet ^4, 5^.

*Akaike information criterion*

We calculated the Akaike information criterion (AIC) for each model selected by lasso regression with ten-fold cross-validation. The AIC is a measure of how well a model fits the data relative to the other possible models given the data analysed and favours fewer parameters^6^. The model with the lowest AIC is the best model approximating the outcome of interest. AIC can be expressed as:

$$AIC= -2(log likelihood)+2K$$

where *K*= number of model parameters and log-likelihood is a measure of model fit^7^. In this study, as *n*/*K* $\leq40$ for sample size *n* and the model with the largest value of *K*, we used the second-order bias correction version of the AIC:

$${AIC}_{c}= -2\left( log likelihood \right)+2K+ \frac{2K\left( K+1 \right)}{n-K-1}$$

$$= AIC+ \frac{2K(K+1)}{n-K-1}$$

where *n* = sample size, *K*= number of model parameters and log-likelihood is a measure of model fit^7^.

*Predictive performance*

Predictive performance was evaluated using the area under the receiver operating characteristic (ROC) curve. The area under the curve (AUC) measures the extent to which a model's predicted probability agrees with the observed outcome, that is the presence or absence of an event.  The AUC is the probability that a randomly chosen patient with the event is rated/ranked higher than a randomly chosen patient without the event. A model performing no better than random will have an AUC of 0.50. (AUC: 0.9-1 = excellent; 0.8-0.9 = good; 0.7-0.8 = fair; 0.6-0.7 = poor; 0.5-0.6 = fail). The AUC was calculated using the R package ROCR ^8^.

*Pathway analysis*

Biological process pathway analysis was carried out using Gene Ontology and PANTHER ^9^. UniProt accession numbers of proteins corresponding to the peptides selected in the final model were uploaded to http://geneontology.org and all *Homo sapiens* genes in the database were used as a reference list. Fisher's Exact with false discovery rate (FDR) multiple test correction was used for determining pathway significance.

1. Supplementary tables and figures

Supplementary Table 1. A summary of the demographic characteristics for the schizophrenia patients and controls in the training and independent test sets. Note that two controls from Cologne were identified as outliers and excluded from the analysis (see Supplementary Methods) and not included in this table. Mean (standard deviation) reported for Age and Body Mass Index (BMI).

| Training set (Cologne) | | | | |
| --- | --- | --- | --- | --- |
|  | First-onset drug-naïve schizophrenia patients | | Controls | |
|  | Males | Females | Males | Females |
| *n* | 31 | 29 | 43 | 34 |
| Age in years | 30.1 (10.2) | 31.8 (10.8) | 31.1 (8.6) | 32.7 (9.0) |
| Smoking (yes/no) | 14/10  (7 missing) | 12/10  (7 missing) | 12/31 | 10/24 |
| Cannabis (yes/no) | 15/8  (8 missing) | 9/14  (7 missing) | 14/29 | 12/19  (3 missing) |
| BMI | 25.0 (5.7)  (3 missing) | 23.8 (5.4)  (3 missing) | 24.0 (3.2)  (15 missing) | 22.3 (3.7)  (9 missing) |
| High school education:  Higher  Middle  Lower  Ongoing | 11  9  8  1  (2 missing) | 19  4  4  0  (2 missing) | 26  3  1  0  (13 missing) | 22  1  0  1  (10 missing) |
|  | | | | |
| Independent test set (Rotterdam) | | | | |
|  | First-onset drug-naïve schizophrenia patients | | Controls | |
|  | Males | Females | Males | Females |
| *n* | 9 | -- | 12 | -- |
| Age in years | 28.3 (5.9) | -- | 28.3 (7.7) | -- |
| Smoking (yes/no) | 6/3 | -- | 2/10 | -- |
| Cannabis (yes/no) | 2/7 | -- | 4/8 | -- |
| BMI | 23.3 (4.7) | -- | 23.9 (1.7) | -- |

Supplementary Table 2. A summary of the 100 models selected using lasso regression with repeated cross-validation. Model selection was conducted in the training set. The eight features used in the two prediction models (Table 1) are shown bold. Estimates of the relative model importance made by summing the Akaike weights across models where the same set of features selected. Note that the Akaike weights across all models sum to one.

| Selected models | Number of features | Frequency | Model probability |
| --- | --- | --- | --- |
| **ANT3/LPGIVAEGR** + **APOA2/SPELQAEAK** + **APOA4/IDQNVEELK** + **APOC3/GWVTDGFSSLK** + **APOH/EHSSLAFWK** + **HPT/VTSIQDWVQK** + **IC1/TNLESILSYPK** + **ITIH4/GPDVLTATVSGK** | 8 | 51 | 0.801 |
| **APOA2/SPELQAEAK** + **APOA4/IDQNVEELK** + **APOC3/GWVTDGFSSLK** + **HPT/VTSIQDWVQK** + **IC1/TNLESILSYPK** + **ITIH4/GPDVLTATVSGK** | 6 | 16 | 0.0270 |
| **ANT3/LPGIVAEGR** + **APOA2/SPELQAEAK** + **APOA4/IDQNVEELK** + **APOC3/GWVTDGFSSLK** + **HPT/VTSIQDWVQK** + **IC1/TNLESILSYPK** + **ITIH4/GPDVLTATVSGK** | 7 | 8 | 0.0159 |
| **APOA4/IDQNVEELK** + **APOC3/GWVTDGFSSLK** + **HPT/VTSIQDWVQK** | 3 | 7 | 0.00241 |
| **APOA2/SPELQAEAK** + **APOA4/IDQNVEELK** + **APOC3/GWVTDGFSSLK** + **HPT/VTSIQDWVQK** + **IC1/TNLESILSYPK** | 5 | 6 | 0.00415 |
| **APOA4/IDQNVEELK** + **APOC3/GWVTDGFSSLK** + **HPT/VTSIQDWVQK** + **IC1/TNLESILSYPK** | 4 | 4 | 0.00173 |
| **ANT3/LPGIVAEGR** + **APOA2/SPELQAEAK** + **APOA4/IDQNVEELK** + **APOC3/GWVTDGFSSLK** + **APOH/EHSSLAFWK** + FCN3/YGIDWASGR + FETUA/HTLNQIDEVK + **HPT/VTSIQDWVQK** + **IC1/TNLESILSYPK** + **ITIH4/GPDVLTATVSGK** | 10 | 4 | 0.123 |
| Age + A2AP/DFLQSLK + A2AP/DSFHLDEQFTVPVEMMQAR + A2AP/FDPSLTQR + A2MG/NEDSLVFVQTDK + **ANT3/LPGIVAEGR** + **APOA2/SPELQAEAK** + **APOA4/IDQNVEELK** + APOC3/DALSSVQESQVAQQAR + **APOC3/GWVTDGFSSLK** + APOC4/AWFLESK + APOE/LEEQAQQIR + APOF/SLPTEDCENEK + **APOH/EHSSLAFWK** + APOL1/LNILNNNYK + C1RL/GSEAINAPGDNPAK + CAH1/ADGLAVIGVLMK + CD5L/EATLQDCPSGPWGK + A2AP/DFLQSLK4 + CO6/TLNICEVGTIR + FCN3/YGIDWASGR + FETUA/HTLNQIDEVK + A2AP/FDPSLTQR6 + **HPT/VTSIQDWVQK** + **IC1/TNLESILSYPK** + IGHA1/DASGVTFTWTPSSGK + IGHA2/DASGATFTWTPSSGK + IGHG2/GLPAPIEK + ITIH4/ETLFSVMPGLK + **ITIH4/GPDVLTATVSGK** + KNG1/DFVQPPTK + RET4/YWGVASFLQK + TTHY/VLDAVR | 33 | 1 | 9.35x10^-10^ |
| Age + A2AP/DSFHLDEQFTVPVEMMQAR + A2AP/FDPSLTQR + A2MG/NEDSLVFVQTDK + **ANT3/LPGIVAEGR** + **APOA2/SPELQAEAK** + **APOA4/IDQNVEELK** + APOC3/DALSSVQESQVAQQAR + **APOC3/GWVTDGFSSLK** + **APOH/EHSSLAFWK** + CAH1/ADGLAVIGVLMK + A2AP/DFLQSLK4 + FCN3/YGIDWASGR + FETUA/HTLNQIDEVK + **HPT/VTSIQDWVQK** + **IC1/TNLESILSYPK** + IGHA1/DASGVTFTWTPSSGK + IGHG2/GLPAPIEK + **ITIH4/GPDVLTATVSGK** + RET4/YWGVASFLQK + AACT/EQLSLLDR | 21 | 1 | 3.99x10^-5^ |
| Age + A2AP/DSFHLDEQFTVPVEMMQAR + A2AP/FDPSLTQR + A2MG/NEDSLVFVQTDK + **ANT3/LPGIVAEGR** + **APOA2/SPELQAEAK** + **APOA4/IDQNVEELK** + APOC3/DALSSVQESQVAQQAR + **APOC3/GWVTDGFSSLK** + **APOH/EHSSLAFWK** + CAH1/ADGLAVIGVLMK + A2AP/DFLQSLK4 + FCN3/YGIDWASGR + FETUA/HTLNQIDEVK + **HPT/VTSIQDWVQK** + **IC1/TNLESILSYPK** + IGHA1/DASGVTFTWTPSSGK + IGHG2/GLPAPIEK + **ITIH4/GPDVLTATVSGK** + RET4/YWGVASFLQK | 20 | 1 | 2.70x10^-5^ |
| **ANT3/LPGIVAEGR** + **APOA2/SPELQAEAK** + **APOA4/IDQNVEELK** + **APOC3/GWVTDGFSSLK** + **APOH/EHSSLAFWK** + FCN3/YGIDWASGR + FETUA/HTLNQIDEVK + **HPT/VTSIQDWVQK** + **IC1/TNLESILSYPK** + IGHA1/DASGVTFTWTPSSGK + **ITIH4/GPDVLTATVSGK** | 11 | 1 | 0.0250 |

Supplementary Table 3. A summary of the 33 features selected using lasso regression with repeated cross-validation. Model selection was conducted in the training set. The eight features used in the two prediction models (Table 1) are shown bold. Estimates of the relative feature importance made by summing the Akaike weights across the subset of models where the feature selected ^10^. Note that the Akaike weights across all models sum to one.

| Protein | Peptide | Relative feature importance | Inclusion fraction |
| --- | --- | --- | --- |
| **HPT** | **VTSIQDWVQK** | **1.000** | **1.00** |
| **APOA4** | **IDQNVEELK** | **1.000** | **1.00** |
| **APOC3** | **GWVTDGFSSLK** | **1.000** | **1.00** |
| **IC1** | **TNLESILSYPK** | **0.998** | **0.93** |
| **APOA2** | **SPELQAEAK** | **0.996** | **0.89** |
| **ITIH4** | **GPDVLTATVSGK** | **0.992** | **0.83** |
| **ANT3** | **LPGIVAEGR** | **0.965** | **0.67** |
| **APOH** | **EHSSLAFWK** | **0.949** | **0.59** |
| FCN3 | YGIDWASGR | 0.148 | 0.08 |
| FETUA | HTLNQIDEVK | 0.148 | 0.08 |
| IGHA1 | DASGVTFTWTPSSGK | 0.025 | 0.04 |
| A2MG | NEDSLVFVQTDK | 6.69E-05 | 0.03 |
| IGHG2 | GLPAPIEK | 6.69E-05 | 0.03 |
| RET4 | YWGVASFLQK | 6.69E-05 | 0.03 |
| APOC3 | DALSSVQESQVAQQAR | 6.69E-05 | 0.03 |
| CAH1 | ADGLAVIGVLMK | 6.69E-05 | 0.03 |
| CLUS | IDSLLENDR | 6.69E-05 | 0.03 |
| A2AP | DSFHLDEQFTVPVEMMQAR | 6.69E-05 | 0.03 |
| A2AP | FDPSLTQR | 6.69E-05 | 0.03 |
| TTHY | VLDAVR | 3.99E-05 | 0.02 |
| IGHA2 | DASGATFTWTPSSGK | 9.35E-10 | 0.01 |
| ITIH4 | ETLFSVMPGLK | 9.35E-10 | 0.01 |
| KNG1 | DFVQPPTK | 9.35E-10 | 0.01 |
| APOC4 | AWFLESK | 9.35E-10 | 0.01 |
| APOE | LEEQAQQIR | 9.35E-10 | 0.01 |
| APOF | SLPTEDCENEK | 9.35E-10 | 0.01 |
| APOL1 | LNILNNNYK | 9.35E-10 | 0.01 |
| C1RL | GSEAINAPGDNPAK | 9.35E-10 | 0.01 |
| CD5L | EATLQDCPSGPWGK | 9.35E-10 | 0.01 |
| A2AP | DFLQSLK | 9.35E-10 | 0.01 |
| CO6 | TLNICEVGTIR | 9.35E-10 | 0.01 |
| HBG1 | MVTAVASALSSR | 9.35E-10 | 0.01 |

Supplementary Table 4. A summary of the 100 models selected using glinternet with repeated cross-validation. Model selection was conducted in the training set. The eight features used in the two prediction models based on lasso regression (Table 1) are shown bold. Estimates of the relative model importance made by summing the Akaike weights across models where the same set of features selected. Note that the Akaike weights across all models sum to one.

| Selected models | Number of features and interactions | Frequency | Model probability |
| --- | --- | --- | --- |
| *Main effects:*  Sex + A2AP/DFLQSLK + **ANT3/LPGIVAEGR** + **APOA2/SPELQAEAK** + **APOA4/IDQNVEELK** + APOC3/DALSSVQESQVAQQAR + **APOC3/GWVTDGFSSLK** + APOE/LEEQAQQIR + **APOH/EHSSLAFWK** + FCN3/YGIDWASGR + FETUA/HTLNQIDEVK + HBA/MFLSFPTTK + HBG1/MVTAVASALSSR + **HPT/VTSIQDWVQK** + **IC1/TNLESILSYPK** + **ITIH4/GPDVLTATVSGK** + SHBG/IALGGLLFPASNLR  *First-order interactions:*  Sex.A2AP/DFLQSLK + Sex.APOE/LEEQAQQIR + Sex.HBA/MFLSFPTTK + Sex.HBG1/MVTAVASALSSR + Sex.SHBG/IALGGLLFPASNLR + | 18 | 49 | 0.231 |
| *Main effects:*  Sex + age + A2AP/DFLQSLK + **ANT3/LPGIVAEGR** + **APOA2/SPELQAEAK** + **APOA4/IDQNVEELK** + APOC3/DALSSVQESQVAQQAR + **APOC3/GWVTDGFSSLK** + APOE/LEEQAQQIR + **APOH/EHSSLAFWK** + FCN3/YGIDWASGR + FETUA/HTLNQIDEVK + HBA/MFLSFPTTK + HBG1/MVTAVASALSSR + **HPT/VTSIQDWVQK** + **IC1/TNLESILSYPK** + IGHG2/GLPAPIEK + **ITIH4/GPDVLTATVSGK** + SHBG/IALGGLLFPASNLR  *First-order interactions:*  Sex.A2AP/DFLQSLK + Sex.APOE/LEEQAQQIR + Sex.HBA/MFLSFPTTK + Sex.HBG1/MVTAVASALSSR + Sex.IGHG2/GLPAPIEK + Sex.SHBG/IALGGLLFPASNLR + | 20 | 31 | 0.016 |
| *Main effects:*  Sex + age + A1BG/ATWSGAVLAGR + A2AP/DFLQSLK + **ANT3/LPGIVAEGR** + **APOA2/SPELQAEAK** + **APOA4/IDQNVEELK** + **APOC3/DALSSVQESQVAQQAR** + APOC3/GWVTDGFSSLK + APOE/LEEQAQQIR + **APOH/EHSSLAFWK** + FCN3/YGIDWASGR + FETUA/HTLNQIDEVK + HBA/MFLSFPTTK + HBG1/MVTAVASALSSR + **HPT/VTSIQDWVQK** + **IC1/TNLESILSYPK** + IGHA1/DASGVTFTWTPSSGK + IGHG2/GLPAPIEK + **ITIH4/GPDVLTATVSGK** + SHBG/IALGGLLFPASNLR  *First-order interactions:*  Sex.A1BG/ATWSGAVLAGR + Sex.A2AP/DFLQSLK + Sex.APOE/LEEQAQQIR + Sex.HBA/MFLSFPTTK + Sex.HBG1/MVTAVASALSSR + Sex.IGHG2/GLPAPIEK + Sex.SHBG/IALGGLLFPASNLR + | 22 | 16 | 0.000688 |
| *Main effects:*  Sex + age + A1BG/ATWSGAVLAGR + A2AP/FDPSLTQR + **ANT3/LPGIVAEGR** + **APOA2/SPELQAEAK** + **APOA4/IDQNVEELK** + APOC3/DALSSVQESQVAQQAR + **APOC3/GWVTDGFSSLK** + APOE/LEEQAQQIR + **APOH/EHSSLAFWK** + CAH1/ADGLAVIGVLMK + FCN3/YGIDWASGR + FETUA/HTLNQIDEVK + HBA/MFLSFPTTK + HBG1/MVTAVASALSSR + **HPT/VTSIQDWVQK** + **IC1/TNLESILSYPK** + IGHA1/DASGVTFTWTPSSGK + IGHG2/GLPAPIEK + **ITIH4/GPDVLTATVSGK** + SHBG/IALGGLLFPASNLR  *First-order interactions:*  Sex.A1BG/ATWSGAVLAGR + Sex.APOE/LEEQAQQIR + Sex.HBA/MFLSFPTTK + Sex.HBG1/MVTAVASALSSR + Sex.IGHG2/GLPAPIEK + Sex.SHBG/IALGGLLFPASNLR + | 23 | 2 | 0.000729 |
| *Main effects:*  Sex + A2AP/DFLQSLK + **ANT3/LPGIVAEGR** + **APOA2/SPELQAEAK** + **APOA4/IDQNVEELK** + APOC3/DALSSVQESQVAQQAR + **APOC3/GWVTDGFSSLK** + APOE/LEEQAQQIR + **APOH/EHSSLAFWK** + HPT/VTSIQDWVQK + **IC1/TNLESILSYPK** + **ITIH4/GPDVLTATVSGK** + SHBG/IALGGLLFPASNLR  *First-order interactions:*  Sex.A2AP/DFLQSLK + Sex.APOE/LEEQAQQIR + Sex.SHBG/IALGGLLFPASNLR + | 14 | 2 | 0.752 |

Supplementary Table 5. A summary of the 77 proteins (147 peptides) available for analysis.

| Uniprot | Protein | Peptide |
| --- | --- | --- |
| P01009 | A1AT | LSITGTYDLK |
| P01009 | A1AT | SPLFMGK |
| P01009 | A1AT | SVLGQLGITK |
| P04217 | A1BG | ATWSGAVLAGR |
| P04217 | A1BG | CLAPLEGAR |
| P04217 | A1BG | SGLSTGWTQLSK |
| P08697 | A2AP | DFLQSLK |
| P08697 | A2AP | DSFHLDEQFTVPVEMMQAR |
| P08697 | A2AP | FDPSLTQR |
| P01023 | A2MG | AIGYLNTGYQR |
| P01023 | A2MG | NEDSLVFVQTDK |
| P01011 | AACT | ADLSGITGAR |
| P01011 | AACT | EIGELYLPK |
| P01011 | AACT | EQLSLLDR |
| P02768 | ALBU | AAFTECCQAADK |
| P02768 | ALBU | ETYGEMADCCAK |
| P02768 | ALBU | QNCELFEQLGEYK |
| P02760 | AMBP | ETLLQDFR |
| P02760 | AMBP | TVAACNLPIVR |
| P01019 | ANGT | ALQDQLVLVAAK |
| P01019 | ANGT | FMQAVTGWK |
| P01019 | ANGT | SLDFTELDVAAEK |
| P01008 | ANT3 | FDTISEK |
| P01008 | ANT3 | LPGIVAEGR |
| P02647 | APOA1 | ATEHLSTLSEK |
| P02647 | APOA1 | EQLGPVTQEFWDNLEK |
| P02652 | APOA2 | SPELQAEAK |
| P06727 | APOA4 | ALVQQMEQLR |
| P06727 | APOA4 | IDQNVEELK |
| P06727 | APOA4 | ISASAEELR |
| P02654 | APOC1 | EFGNTLEDK |
| P02654 | APOC1 | EWFSETFQK |
| P02655 | APOC2 | ESLSSYWESAK |
| P02655 | APOC2 | TAAQNLYEK |
| P02656 | APOC3 | DALSSVQESQVAQQAR |
| P02656 | APOC3 | GWVTDGFSSLK |
| P55056 | APOC4 | AWFLESK |
| P05090 | APOD | VLNQELR |
| P02649 | APOE | AATVGSLAGQPLQER |
| P02649 | APOE | ALMDETMK |
| P02649 | APOE | LEEQAQQIR |
| P02649 | APOE | LGPLVEQGR |
| P02649 | APOE | SELEEQLTPVAEETR |
| Q13790 | APOF | SLPTEDCENEK |
| P02749 | APOH | EHSSLAFWK |
| P02749 | APOH | VSFFCK |
| O14791 | APOL1 | LNILNNNYK |
| O14791 | APOL1 | VNEPSILEMSR |
| O14791 | APOL1 | VTEPISAESGEQVER |
| O95445 | APOM | AFLLTPR |
| O95445 | APOM | SLTSCLDSK |
| P02747 | C1QC | TNQVNSGGVLLR |
| P00736 | C1R | YTTEIIK |
| Q9NZP8 | C1RL | GSEAINAPGDNPAK |
| P09871 | C1S | LLEVPEGR |
| P09871 | C1S | TNFDNDIALVR |
| P04003 | C4BPA | EDVYVVGTVLR |
| P04003 | C4BPA | FSAICQGDGTWSPR |
| P04003 | C4BPA | YTCLPGYVR |
| P00915 | CAH1 | ADGLAVIGVLMK |
| P08185 | CBG | GTWTQPFDLASTR |
| P08185 | CBG | ITQDAQLK |
| Q96IY4 | CBPB2 | DTGTYGFLLPER |
| Q96IY4 | CBPB2 | YPLYVLK |
| O43866 | CD5L | EATLQDCPSGPWGK |
| P00450 | CERU | EVGPTNADPVCLAK |
| P00450 | CERU | NNEGTYYSPNYNPQSR |
| P00751 | CFAB | DISEVVTPR |
| P00751 | CFAB | DLLYIGK |
| P00751 | CFAB | EELLPAQDIK |
| P00751 | CFAB | YGLVTYATYPK |
| P08603 | CFAH | CFEGFGIDGPAIAK |
| P10909 | CLUS | FMETVAEK |
| P10909 | CLUS | IDSLLENDR |
| P06681 | CO2 | HAIILLTDGK |
| P01024 | CO3 | AGDFLEANYMNLQR |
| P01024 | CO3 | VYAYYNLEESCTR |
| P0C0L4 | CO4A | DFALLSLQVPLK |
| P0C0L4 | CO4A | ITQVLHFTK |
| P0C0L4 | CO4A | VLSLAQEQVGGSPEK |
| P13671 | CO6 | SEYGAALAWEK |
| P13671 | CO6 | TLNICEVGTIR |
| P07357 | CO8A | MESLGITSR |
| P02748 | CO9 | LSPIYNLVPVK |
| P02748 | CO9 | VVEESELAR |
| P00748 | FA12 | CFEPQLLR |
| P00748 | FA12 | VVGGLVALR |
| O75636 | FCN3 | YGIDWASGR |
| P02765 | FETUA | FSVVYAK |
| P02765 | FETUA | HTLNQIDEVK |
| P02751 | FINC | YSFCTDHTVLVQTR |
| P06396 | GELS | AGALNSNDAFVLK |
| P06396 | GELS | SEDCFILDHGK |
| P69905 | HBA | FLASVSTVLTSK |
| P69905 | HBA | MFLSFPTTK |
| P69891 | HBG1 | MVTAVASALSSR |
| P02790 | HEMO | NFPSPVDAAFR |
| P02790 | HEMO | VDGALCMEK |
| P05546 | HEP2 | FAFNLYR |
| P05546 | HEP2 | IAIDLFK |
| P00738 | HPT | DYAEVGR |
| P00738 | HPT | VGYVSGWGR |
| P00738 | HPT | VTSIQDWVQK |
| P04196 | HRG | ADLFYDVEALDLESPK |
| P04196 | HRG | DSPVLIDFFEDTER |
| P05155 | IC1 | FQPTLLTLPR |
| P05155 | IC1 | TNLESILSYPK |
| P01876 | IGHA1 | DASGVTFTWTPSSGK |
| P01876 | IGHA1 | TPLTATLSK |
| P01877 | IGHA2 | DASGATFTWTPSSGK |
| P01857 | IGHG1 | FNWYVDGVEVHNAK |
| P01859 | IGHG2 | GLPAPIEK |
| P01859 | IGHG2 | TTPPMLDSDGSFFLYSK |
| P01860 | IGHG3 | DTLMISR |
| P01860 | IGHG3 | NQVSLTCLVK |
| P01871 | IGHM | QIQVSWLR |
| P01871 | IGHM | YAATSQVLLPSK |
| P19827 | ITIH1 | GSLVQASEANLQAAQDFVR |
| P19827 | ITIH1 | LDAQASFLPK |
| P19823 | ITIH2 | FYNQVSTPLLR |
| P19823 | ITIH2 | IQPSGGTNINEALLR |
| Q14624 | ITIH4 | ETLFSVMPGLK |
| Q14624 | ITIH4 | GPDVLTATVSGK |
| P03952 | KLKB1 | LSMDGSPTR |
| P01042 | KNG1 | DFVQPPTK |
| P01042 | KNG1 | DIPTNSPELEETLTHTITK |
| P51884 | LUM | SLEDLQLTHNK |
| P36955 | PEDF | DTDTGALLFIGK |
| P36955 | PEDF | ELLDTVTAPQK |
| P36955 | PEDF | LQSLFDSPDFSK |
| P36955 | PEDF | TVQAVLTVPK |
| Q96PD5 | PGRP2 | GCPDVQASLPDAK |
| Q96PD5 | PGRP2 | TFTLLDPK |
| P80108 | PHLD | NQVVIAAGR |
| P00747 | PLMN | FVTWIEGVMR |
| P02753 | RET4 | QEELCLAR |
| P02753 | RET4 | YWGVASFLQK |
| P02743 | SAMP | IVLGQEQDSYGGK |
| P04278 | SHBG | IALGGLLFPASNLR |
| P05452 | TETN | EQQALQTVCLK |
| P00734 | THRB | ELLESYIDGR |
| P00734 | THRB | SGIECQLWR |
| P02787 | TRFE | EGYYGYTGAFR |
| P02766 | TTHY | AADDTWEPFASGK |
| P02766 | TTHY | VLDAVR |
| P04004 | VTNC | DVWGIEGPIDAAFTR |
| P04004 | VTNC | DWHGVPGQVDAAMAGR |

Supplementary Table 6. Reported schizophrenia associations for the 77 proteins available for analysis.

| Uniprot | Protein | Schizophrenia |
| --- | --- | --- |
| P01009 | A1AT |  |
| P04217 | A1BG |  |
| P08697 | A2AP | ^11^ |
| P01023 | A2MG |  |
| P01011 | AACT | ^12, 13^ |
| P02768 | ALBU | ^14-16^ |
| P02760 | AMBP | ^17-19^ |
| P01019 | ANGT |  |
| P01008 | ANT3 | ^11, 20^ |
| P02647 | APOA1 | ^11, 14, 19, 21-24^ |
| P02652 | APOA2 | ^11, 24, 25^ |
| P06727 | APOA4 | ^11, 22, 25, 26^ |
| P02654 | APOC1 | ^11, 25, 27^ |
| P02655 | APOC2 |  |
| P02656 | APOC3 | ^11^ |
| P55056 | APOC4 |  |
| P05090 | APOD | ^11, 25, 28, 29^ |
| P02649 | APOE | ^11, 23, 30^ |
| Q13790 | APOF |  |
| P02749 | APOH | ^31^ |
| O14791 | APOL1 |  |
| O95445 | APOM |  |
| P02747 | C1QC |  |
| P00736 | C1R |  |
| Q9NZP8 | C1RL |  |
| P09871 | C1S |  |
| P04003 | C4BPA | ^32^ |
| P00915 | CAH1 |  |
| P08185 | CBG |  |
| Q96IY4 | CBPB2 |  |
| O43866 | CD5L |  |
| P00450 | CERU | ^33-35^ |
| P00751 | CFAB | ^24^ |
| P08603 | CFAH |  |
| P10909 | CLUS | ^27, 36, 37^ |
| P06681 | CO2 |  |
| P01024 | CO3 |  |
| P0C0L4 | CO4A | ^38^ |
| P13671 | CO6 | ^24^ |
| P07357 | CO8A |  |
| P02748 | CO9 | ^24^ |
| P00748 | FA12 |  |
| O75636 | FCN3 | ^24^ |
| P02765 | FETUA |  |
| P02751 | FINC |  |
| P06396 | GELS |  |
| P69905 | HBA |  |
| P69891 | HBG1 |  |
| P02790 | HEMO | ^26^ |
| P05546 | HEP2 |  |
| P00738 | HPT | ^22, 26, 31, 33, 39-42^ |
| P04196 | HRG |  |
| P05155 | IC1 |  |
| P01876 | IGHA1 |  |
| P01877 | IGHA2 |  |
| P01857 | IGHG1 |  |
| P01859 | IGHG2 |  |
| P01860 | IGHG3 | ^43^ |
| P01871 | IGHM |  |
| P19827 | ITIH1 |  |
| P19823 | ITIH2 | ^44^ |
| Q14624 | ITIH4 | ^37, 45^ |
| P03952 | KLKB1 | ^11^ |
| P01042 | KNG1 |  |
| P51884 | LUM |  |
| P36955 | PEDF |  |
| Q96PD5 | PGRP2 |  |
| P80108 | PHLD |  |
| P00747 | PLMN |  |
| P02753 | RET4 |  |
| P02743 | SAMP | ^21^ |
| P04278 | SHBG | ^46^ |
| P05452 | TETN |  |
| P00734 | THRB |  |
| P02787 | TRFE |  |
| P02766 | TTHY | ^22^ |
| P04004 | VTNC |  |

Supplementary Figure 1. A plot of the first two principle components for the Cologne study. The first principle component identifies two outlying control samples based on their relative peptide abundance.

Supplementary Figure 2. Coefficients of variation calculated for the pooled plate 1 samples, included on plates 1 (seven sample injections) and 2 (four sample injections) of the Cologne study. The median CV was 5.01% (5.49% in plate 1 and 4.53% in plate2) based upon 77 proteins (147 peptides).

Supplementary Figure 3. Boxplots of abundance for the 16 protein-peptides selected in the glinternet model selection (Table 3) by sex. Note that SHBG, APOE, A2AP, HBA and HBG1 had first-order interactions with sex.

3.0 References

1. Ozcan S, Cooper JD, Lago SG, Kenny D, Rustogi N, Stocki P *et al.* Towards reproducible MRM based biomarker discovery using dried blood spots. *Sci Rep* 2017; **7:** 45178.

2. Dudoit S, Yang YH, Callow MJ, Speed TP. Statistical methods for identifying differentially expressed genes in replicated cDNA microarray experiments. *Stat Sinica* 2002; **12**(1)**:** 111-139.

3. Schiff MH, Jaffe JS, Freundlich B. Head-to-head, randomised, crossover study of oral versus subcutaneous methotrexate in patients with rheumatoid arthritis: drug-exposure limitations of oral methotrexate at doses >/=15 mg may be overcome with subcutaneous administration. *Ann Rheum Dis* 2014; **73**(8)**:** 1549-1551.

4. Tibshirani R. Regression shrinkage and selection via the Lasso. *J Roy Stat Soc B Met* 1996; **58**(1)**:** 267-288.

5. Hastie T, Tibshirani R, Friedman J. *The Elements of Statistical Learning: Data Mining, Inference, and Prediction*. 5th Edition edn. Springer, New York, NY, USA2001.

6. Akaike H. *Information theory as anextension of the maximum likelihood principle.* Budapest, Akaemiai Kiado1973.

7. Burnham KP, Anderson DR. Multimodel Inference: understanding AIC and BIC in model selection. *Sociological Methods and Research* 2004; **33**(2)**:** 261-304.

8. Sing T, Sander O, Beerenwinkel N, Lengauer T. ROCR: visualizing classifier performance in R. *Bioinformatics* 2005; **21**(20)**:** 3940-3941.

9. Mi H, Huang X, Muruganujan A, Tang H, Mills C, Kang D *et al.* PANTHER version 11: expanded annotation data from Gene Ontology and Reactome pathways, and data analysis tool enhancements. *Nucleic Acids Res* 2017; **45**(D1)**:** D183-D189.

10. Burnham KP, Anderson DR. *Model selection and multimodel inference*. 2nd edn. Springer: New York, 2002.

11. Knochel C, Kniep J, Cooper JD, Stablein M, Wenzler S, Sarlon J *et al.* Altered apolipoprotein C expression in association with cognition impairments and hippocampus volume in schizophrenia and bipolar disorder. *European archives of psychiatry and clinical neuroscience* 2016.

12. Tomasik J, Rahmoune H, Guest PC, Bahn S. Neuroimmune biomarkers in schizophrenia. *Schizophrenia research* 2016; **176**(1)**:** 3-13.

13. Fillman SG, Cloonan N, Catts VS, Miller LC, Wong J, McCrossin T *et al.* Increased inflammatory markers identified in the dorsolateral prefrontal cortex of individuals with schizophrenia. *Molecular psychiatry* 2013; **18**(2)**:** 206-214.

14. Huang TL. Decreased serum albumin levels in Taiwanese patients with schizophrenia. *Psychiatry and clinical neurosciences* 2002; **56**(6)**:** 627-630.

15. Babushkina TA, Klimova TP, Peregudov AS, Gryzunov YA, Smolina NV, Dobretsov GE *et al.* Study of high-resolution H1 nuclear magnetic resonance spectra of the serum and its albumin faction in patients with the first schizophrenia episode. *Bulletin of experimental biology and medicine* 2012; **152**(6)**:** 748-751.

16. Reddy R, Keshavan M, Yao JK. Reduced plasma antioxidants in first-episode patients with schizophrenia. *Schizophrenia research* 2003; **62**(3)**:** 205-212.

17. Viana J, Hannon E, Dempster E, Pidsley R, Macdonald R, Knox O *et al.* Schizophrenia-associated methylomic variation: molecular signatures of disease and polygenic risk burden across multiple brain regions. *Human molecular genetics* 2016.

18. Wan C, La Y, Zhu H, Yang Y, Jiang L, Chen Y *et al.* Abnormal changes of plasma acute phase proteins in schizophrenia and the relation between schizophrenia and haptoglobin (Hp) gene. *Amino Acids* 2007; **32**(1)**:** 101-108.

19. Schwarz E, Guest PC, Rahmoune H, Martins-de-Souza D, Niebuhr DW, Weber NS *et al.* Identification of a blood-based biological signature in subjects with psychiatric disorders prior to clinical manifestation. *World J Biol Psychiatry* 2012; **13**(8)**:** 627-632.

20. Carrizo E, Fernandez V, Quintero J, Connell L, Rodriguez Z, Mosquera M *et al.* Coagulation and inflammation markers during atypical or typical antipsychotic treatment in schizophrenia patients and drug-free first-degree relatives. *Schizophr Res* 2008; **103**(1-3)**:** 83-93.

21. La YJ, Wan CL, Zhu H, Yang YF, Chen YS, Pan YX *et al.* Decreased levels of apolipoprotein A-I in plasma of schizophrenic patients. *Journal of neural transmission* 2007; **114**(5)**:** 657-663.

22. Yang Y, Wan C, Li H, Zhu H, La Y, Xi Z *et al.* Altered levels of acute phase proteins in the plasma of patients with schizophrenia. *Anal Chem* 2006; **78**(11)**:** 3571-3576.

23. Martins-De-Souza D, Wobrock T, Zerr I, Schmitt A, Gawinecka J, Schneider-Axmann T *et al.* Different apolipoprotein E, apolipoprotein A1 and prostaglandin-H2 D-isomerase levels in cerebrospinal fluid of schizophrenia patients and healthy controls. *World J Biol Psychiatry* 2010; **11**(5)**:** 719-728.

24. Jaros JA, Martins-de-Souza D, Rahmoune H, Rothermundt M, Leweke FM, Guest PC *et al.* Protein phosphorylation patterns in serum from schizophrenia patients and healthy controls. *Journal of proteomics* 2012; **76 Spec No.:** 43-55.

25. Levin Y, Wang L, Schwarz E, Koethe D, Leweke FM, Bahn S. Global proteomic profiling reveals altered proteomic signature in schizophrenia serum. *Mol Psychiatry* 2010; **15**(11)**:** 1088-1100.

26. Jiang L, Lindpaintner K, Li HF, Gu NF, Langen H, He L *et al.* Proteomic analysis of the cerebrospinal fluid of patients with schizophrenia. *Amino acids* 2003; **25**(1)**:** 49-57.

27. Schwarz E, Guest PC, Steiner J, Bogerts B, Bahn S. Identification of blood-based molecular signatures for prediction of response and relapse in schizophrenia patients. *Transl Psychiatry* 2012; **2:** e82.

28. Thomas EA, Dean B, Pavey G, Sutcliffe JG. Increased CNS levels of apolipoprotein D in schizophrenic and bipolar subjects: implications for the pathophysiology of psychiatric disorders. *Proceedings of the National Academy of Sciences of the United States of America* 2001; **98**(7)**:** 4066-4071.

29. Mahadik SP, Khan MM, Evans DR, Parikh VV. Elevated plasma level of apolipoprotein D in schizophrenia and its treatment and outcome. *Schizophrenia research* 2002; **58**(1)**:** 55-62.

30. Dean B, Laws SM, Hone E, Taddei K, Scarr E, Thomas EA *et al.* Increased levels of apolipoprotein E in the frontal cortex of subjects with schizophrenia. *Biological psychiatry* 2003; **54**(6)**:** 616-622.

31. Chan MK, Krebs MO, Cox D, Guest PC, Yolken RH, Rahmoune H *et al.* Development of a blood-based molecular biomarker test for identification of schizophrenia before disease onset. *Transl Psychiatry* 2015; **5:** e601.

32. Sainz J, Mata I, Barrera J, Perez-Iglesias R, Varela I, Arranz MJ *et al.* Inflammatory and immune response genes have significantly altered expression in schizophrenia. *Molecular psychiatry* 2013; **18**(10)**:** 1056-1057.

33. Wong CT, Tsoi WF, Saha N. Acute phase proteins in male Chinese schizophrenic patients in Singapore. *Schizophrenia research* 1996; **22**(2)**:** 165-171.

34. Wolf TL, Kotun J, Meador-Woodruff JH. Plasma copper, iron, ceruloplasmin and ferroxidase activity in schizophrenia. *Schizophrenia research* 2006; **86**(1-3)**:** 167-171.

35. Virit O, Selek S, Bulut M, Savas HA, Celik H, Erel O *et al.* High ceruloplasmin levels are associated with obsessive compulsive disorder: a case control study. *Behavioral and brain functions : BBF* 2008; **4:** 52.

36. Shastri A, Bonifati DM, Kishore U. Innate immunity and neuroinflammation. *Mediators of inflammation* 2013; **2013:** 342931.

37. Athanas KM, Mauney SL, Woo TU. Increased extracellular clusterin in the prefrontal cortex in schizophrenia. *Schizophrenia research* 2015; **169**(1-3)**:** 381-385.

38. Sekar A, Bialas AR, de Rivera H, Davis A, Hammond TR, Kamitaki N *et al.* Schizophrenia risk from complex variation of complement component 4. *Nature* 2016; **530**(7589)**:** 177-183.

39. Johnson G, Brane D, Block W, van Kammen DP, Gurklis J, Peters JL *et al.* Cerebrospinal fluid protein variations in common to Alzheimer's disease and schizophrenia. *Applied and theoretical electrophoresis : the official journal of the International Electrophoresis Society* 1992; **3**(2)**:** 47-53.

40. Maes M, Delanghe J, Bocchio Chiavetto L, Bignotti S, Tura GB, Pioli R *et al.* Haptoglobin polymorphism and schizophrenia: genetic variation on chromosome 16. *Psychiatry research* 2001; **104**(1)**:** 1-9.

41. Rudduck C, Franzen G, Frohlander N, Lindstrom L. Haptoglobin and transferrin types in schizophrenia. *Human heredity* 1985; **35**(2)**:** 65-68.

42. Saha N, Bhattacharyya SP, Tsoi WF. Serum protein markers in schizophrenia: haptoglobin, transferrin and group-specific component types. *Singapore medical journal* 1985; **26**(4-5)**:** 337-340.

43. Pandey JP, Namboodiri AM, Elston RC. Immunoglobulin G genotypes and the risk of schizophrenia. *Human genetics* 2016; **135**(10)**:** 1175-1179.

44. Blomstrom A, Gardner RM, Dalman C, Yolken RH, Karlsson H. Influence of maternal infections on neonatal acute phase proteins and their interaction in the development of non-affective psychosis. *Translational psychiatry* 2015; **5:** e502.

45. Schizophrenia Psychiatric Genome-Wide Association Study C. Genome-wide association study identifies five new schizophrenia loci. *Nat Genet* 2011; **43**(10)**:** 969-976.

46. Ramsey JM, Schwarz E, Guest PC, van Beveren NJ, Leweke FM, Rothermundt M *et al.* Distinct molecular phenotypes in male and female schizophrenia patients. *PLoS One* 2013; **8**(11)**:** e78729.
